# *Streptococcus equi* subsp. *zooepidemicus* Supernatant Containing Streptolysin S Alters the Equine Nasal and Vaginal Mucosa, Modulating Equine Herpesvirus 1, 3 and 4 Infections

**DOI:** 10.3390/v17070980

**Published:** 2025-07-14

**Authors:** Eslam Mohamed, Jolien Van Cleemput, Burak Şahin, Wim Van den Broeck, Filip Boyen, Hans Nauwynck

**Affiliations:** 1 Department of Translational Physiology, Infectiology and Public Health, Faculty of Veterinary Medicine, Ghent University, 9820 Merelbeke, Belgium; jolien.vancleemput@ugent.be (J.V.C.); hans.nauwynck@ugent.be (H.N.); 2 Department of Animal Medicine, Faculty of Veterinary Medicine, Benha University, Moshtohor 13736, Egypt; 3 Department of Virology, Institute of Health Sciences, Burdur Mehmet Akif Ersoy University, 15100 Burdur, Turkey; bsahin1507@gmail.com; 4 Department of Morphology, Medical Imaging, Orthopedics and Nutrition, Faculty of Veterinary Medicine, Ghent University, 9820 Merelbeke, Belgium; wim.vandenbroeck@ugent.be; 5 Department of Pathobiology, Pharmacology and Zoological Medicine, Faculty of Veterinary Medicine, Ghent University, 9820 Merelbeke, Belgium; filip.boyen@ugent.be

**Keywords:** SEZ, SLS toxin, EHV-1, EHV-3, EHV-4, horse mucosal microbiome, horse mucosal explants, bacterial–viral interaction, EGTA

## Abstract

The equine respiratory and reproductive tract microbiomes are complex and subject to constant fluctuations. Among the microbial inhabitants, *Streptococcus equi* subsp. *zooepidemicus* (SEZ) is recognized as the dominant bacterium. It is an opportunistic pathogen that may occasionally lead to various types of infections. A key virulence factor of SEZ is the streptolysin S (SLS) toxin, which is responsible for the characteristic β-hemolysis on blood agar and tissue damage. Viruses and bacteria may interact and aggravate lesions and disease. This study aimed to evaluate the impact of an SLS-containing supernatant from SEZ on the nasal and vaginal mucosa and the subsequent replication of equine herpesviruses. The SLS-containing supernatant was prepared, and three 10-fold dilutions (optical density “OD” 10^−2^, 10^−3^, 10^−4^) were applied to equine nasal and vaginal explants. Untreated and EGTA-treated explants served as controls. Epithelial integrity was assessed by measuring the thickness and intercellular spaces. Nasal explants were inoculated with EHV-1 and EHV-4, while vaginal explants received EHV-1 and EHV-3. Viral replication was estimated via immunofluorescence staining and confocal microscopy. SLS-containing supernatants 10^−2^ and 10^−3^ compromised epithelial integrity. Viral replication increased in explants treated with SLS 10^−3^, demonstrating SLS’s damaging effects on the epithelium, facilitating equine herpesvirus replication.

## 1. Introduction

Equine herpesviruses cause contagious diseases in equids worldwide. Equine herpesvirus type 1 (EHV-1) replicates mainly in the upper respiratory tract, resulting in respiratory disorders. Via viremia, it reaches its secondary target organs, including the pregnant uterus, central nervous system, and eyes. Replication in these organs may lead to abortion and neonatal foal death, equine herpes myeloencephalopathy (EHM), and chorioretinal lesions [1]. Equine herpesvirus type 3 (EHV-3) causes a sexually transmitted disease called equine coital exanthema (ECE). This condition affects mainly the genital areas, leading to the development of papules, vesicles, pustules, and ulcers in the mucosa of the vagina and vestibules in mares, as well as the penis and prepuce in stallions [2]. Equine herpesvirus type 4 (EHV-4) is the second most prevalent herpesvirus affecting the upper respiratory tract in horses [3].

The interplay between host and environmental factors determines the outcomes of viral infections. In this context, the host microbiome plays a pivotal role in shaping the course and development of these infections. In its equilibrium state, the microbiome is thought to provide significant protection to the host. These include strengthening innate barrier function, regulating the immune response, and inhibiting the growth of harmful pathogens [4]. An imbalance in the microbiome (dysbiosis) can directly or indirectly influence the host defense system. In humans, the impact of the microbiota on viral infections has been investigated. Tsang et al. demonstrated that the respiratory microbiome could be a helpful predictor of the susceptibility to influenza A (H3N2) virus at the time of exposure [5]. Moreover, Erickson et al. illustrated that the gut microbiota enhances enteric viruses’ replication, transmission, and clinical outcomes [6]. The study of the bacterial microbiota has gained importance in veterinary medicine, including in the equine respiratory and reproductive systems [7,8]. Recent investigations have consistently identified *Streptococcus equi* subsp. *zooepidemicus* (SEZ) as the most prevalent bacterium in both respiratory and reproductive samples from horses [7]. While SEZ can be frequently detected in healthy horses, it is also the leading cause of various types of infections, including respiratory disease and endometritis [9,10].

SEZ is a Gram-positive, β-hemolytic bacterium classified under Lancefield group C, sharing this classification with *S. equi* subsp. *equi*, *S. equi* subsp. *ruminatorum*, *S. dysgalactiae* subsp. *equisimilis*, and *S. dysgalactiae* subsp. *dysgalactiae* [11]. It is an opportunistic organism that typically colonizes different mucosal surfaces in equids. However, it can cause a range of infections in other animals, such as cattle, sheep, pigs, and chickens [12]. Although rare, SEZ can also cause zoonotic infections in humans, leading to meningitis, pneumonia, endocarditis, septic arthritis, osteomyelitis, myositis, and sepsis [13]. SEZ shares > 98% DNA sequence homology with *S. equi* subsp. *equi* and >80% with the important human pathogen *S. pyogenes* [14]. Moreover, SEZ harbors virulence factors similar to those of *S. equi* subsp. *equi* and *S. pyogenes*. These factors include cytotoxin streptolysin S (SLS), fibronectin-binding factor, M-like protein, immunoglobulin-binding proteins, and various superantigens, as well as the ability to synthesize a hyaluronic acid capsule, all of which contribute to its pathogenicity [14].

SLS, a toxin produced by Lancefield groups A, C, and G streptococci, is a small (~2.7 kDa) and non-immunogenic peptide that undergoes extensive post-translational modifications before secretion. It is encoded by the SLS-associated gene (*sag*) operon, which consists of the nine-gene *sagABCDEFGHI* [15]. The transcription process begins with *sagA*, resulting in the 53-amino-acid SLS precursor. This precursor is post-translationally modified by the *sagBCD*-encoded enzyme complex, transforming it into its mature form. Subsequently, sagE plays a crucial role by cleaving the amino-terminal leader sequence from the mature core peptide. Finally, the modified core peptide is exported out of the cell through the sagGHI complex. SLS has a broad cytolytic range, targeting epithelial cells, erythrocytes, leukocytes, platelets, and their subcellular organelles [16,17].

In over 100 years of research, SLS has been widely studied with *S. pyogenes*, where it has been shown to play a significant role in hemolysis and cytotoxicity, contributing to the pathogen’s virulence in both in vivo and in vitro models. For example, Sumitomo et al. employed human colon carcinoma epithelial (Caco-2) and keratinocyte (HaCaT) cell lines to investigate the role of SLS in disrupting intercellular junctions and promoting the translocation of group A streptococci [18]. An in vivo study in mice by Shannon et al. confirmed that SLS is essential in establishing nasopharyngeal and skin infections by *S. pyogenes* [19].

Bacterial–viral co-infections represent a well-documented phenomenon across veterinary medicine, forming the basis of respiratory disease complexes in cattle, dogs, and pigs [20]. In horses, Gomez et al. demonstrated the variability in the respiratory microbial community structure in EHV-1-infected horses compared to healthy ones [21]. However, there is limited research on SEZ and the role of SLS in its interaction with the horse mucosae, as well as its influence on the progression of viral infections [22]. Therefore, this study aimed to investigate the effects of SLS on the nasal and vaginal mucosae and its potential role in modulating the subsequent replication of EHV-1, EHV-4, and EHV-3 in these mucosae.

## 2. Materials and Methods

### 2.1. Preparation of the Cell-Free Sls-Containing Supernatant

SEZ strain 4069, isolated from a mare’s uterus in 2018 and identified using MALDI-TOF MS [23], was used to prepare the SLS-containing supernatant, as previously described by Yokohata et al. in 2023 [24]. Briefly, 10 µL of the bacteria glycerol stock was cultured on Columbia Agar^®^ with 5% sheep blood (ThermoFischer Scientific, Paisley, UK) and incubated overnight at 37 °C in 5% CO_2_. Subsequently, one colony was transferred to brain heart infusion (BHI) broth (MP Biomedicals, Solon, OH, USA) and incubated at 37 °C overnight with shaking at 125 rounds per minute (rpm) in 5% CO_2_.

The preincubated bacteria were inoculated into the co-cultivation medium (CM) [Dulbecco’s modified eagle’s medium including GlutaMAX (DMEM; ThermoFisher Scientific, Paisley, UK) and Roswell Park Memorial Institute Medium including Glutamax and HEPES (RPMI; ThermoFisher Scientific, Paisley, UK), supplemented with 1.0% (*w*/*v*) of bovine serum albumin (BSA; Nacalai Tesque, Inc., Kyoto, Japan), and 10% (*v*/*v*) of BHI broth] and incubated for 4 h at 37 °C and under 125 rpm shaking in 5% CO_2_. Then, the optical density (OD) was checked to prepare the concentrations used in this study accordingly. The culture supernatant was obtained by centrifugation at 13,000× *g* for 5 min (min) at room temperature (RT). The SLS-containing supernatant was subjected to filtration using a 0.22 µm filter (Merck Millipore, Burlington, MA, United States) and stored at −70 °C. In parallel, the preparation of an SLS-containing supernatant without BSA supplementation was performed.

### 2.2. Assessment of the Hemolytic Activity of the Prepared Cell-Free Sls-Containing Supernatant

To assess the presence of active SLS in the supernatant prepared with BSA, Columbia Agar^®^ with 5% sheep blood (ThermoFischer Scientific) was inoculated and incubated overnight at 37 °C in 5% CO_2_. The presence of active SLS was confirmed by the characteristic β-hemolysis, while the absence of bacterial growth confirmed the sterility of the supernatant [19]. Concurrently, the supernatant prepared without BSA was used as a negative control for the hemolysis and as proof for the crucial role of BSA as a carrier and stabilizer of SLS in the absence of the bacteria. Based on this assessment, we used the filtered SLS-containing supernatant supplemented with BSA in our study.

### 2.3. Isolation and Culturing of Ex Vivo Nasal and Vaginal Explants

Samples of the posterior nasal septum and the middle portion of the vagina were collected from three healthy horses at the slaughterhouse. The tissue was submerged in transport medium consisting of 500 mL of phosphate-buffered saline (PBS) containing calcium and magnesium and supplemented with 100 U/mL penicillin (ThermoFisher Scientific), 0.1 mg/mL streptomycin (ThermoFisher Scientific), 0.1 mg/mL gentamicin (ThermoFisher Scientific), 0.1 mg/mL kanamycin (Merck, Darmstadt, Germany), and 0.25 μg/mL amphotericin B (ThermoFisher Scientific) and transported to the laboratory on ice.

Upon arrival, the mucosae were separated from the underlying tissue and divided into uniform explants measuring 25 mm^2^ using sterile tweezers and a No. 24 surgical blade (Swann-Morton, Sheffield, UK). A single explant per tissue type was embedded in 2% methylcellulose (Methocel^®^; Sigma-Aldrich, St. Louis, MO, USA) and snap-frozen using dry ice and 100% ethanol and stored at −70 °C until processing. The remaining explants were subjected to an air–liquid interface cultivation method for 18 h at 37 °C with 5% CO_2_, as previously described by Vandekerckhove et al. in 2009 for the nasal septum and Negussie et al. in 2016 for the vagina [25,26]. Briefly, explants were positioned onto sterilized fine-meshed gauzes with the epithelium facing upwards. The gauzes were then placed in 6-well plates containing serum-free medium (SFM), composed of a 50% mixture of DMEM (ThermoFisher Scientific) and RPMI, including Glutamax and HEPES (ThermoFisher Scientific), supplemented with 100 U/mL penicillin, 0.1 mg/mL streptomycin, 0.1 mg/mL gentamicin, and 0.25 μg/mL amphotericin B. The explants were maintained at 37 °C and 5% CO_2_.

### 2.4. Pretreatment of the Nasal and Vaginal Explants with the Prepared Bsa-Supplemented Sls-Containing Supernatant

For each tissue, the pretreatment was performed in triplicate. In each replicate, 20 explants (4 per treatment condition) were thoroughly washed and embedded in agarose to mimic the in vivo conditions, as described by Vairo et al. [27]. The apical surface of the embedded explant was treated for 1 h with 1 mL of three concentrations of the prepared SLS-containing supernatant (10^−2^, 10^−3^, 10^−4^), diluted in CM. Additionally, 1 h treatments with CM and 8 mM of the Ca^2+^ chelating agent ethylene glycol tetraacetic acid (EGTA; VWR, Leuven, Belgium) [28] were used as negative and positive controls for the disruption of cell–cell junctions, respectively.

### 2.5. Toxicity Assessment of the Prepared Bsa-Supplemented Sls-Containing Supernatant

After 1 h of treatment, one explant per treatment condition was thoroughly washed twice with SFM, embedded in Methocel^®^, snap-frozen using dry ice and 100% ethanol, and stored at −70 °C until processing. The toxicity of the SLS-containing supernatant was assessed for each treatment using a terminal deoxynucleotidyl transferase dUTP nick end-labeling (TUNEL)-based in situ cell death detection kit, fluorescein (Roche Diagnostics Corporation, Basel, Switzerland). TUNEL-positive cells were counted among 100 epithelial and 100 lamina propria cells at three randomly chosen locations [27].

### 2.6. Assessment of Epithelial Intercellular Integrity

After 1 h of treatment, one explant per treatment condition was thoroughly washed twice with SFM and placed in 3.5% formaldehyde fixative for 24 h. Afterwards, the fixed explants were moved to 70% ethanol until further processing and embedding in paraffin using an automated STP120 Spin Tissue Processor (ThermoFisher Scientific). Next, 8 µm sections of the paraffin-embedded tissue samples were deparaffinized in xylene, rehydrated in descending grades of alcohol, and stained with hematoxylin and eosin (HE). They were then dehydrated in ascending grades of alcohol and xylene and mounted with DPX (Sigma-Aldrich) [28].

Light microscopy (Olympus BX61, Tokyo, Japan) images of each treated explant were taken, and the percentage of the intercellular space and epithelial thickness were assessed using the Fiji software (ImageJ v2.14, National Institutes of Health [NIH], Bethesda, MD, USA) [29]. For each image, the epithelium was manually selected as the region of interest (ROI) using the ROI manager tool v2.14, excluding any completely detached and isolated cells. To measure the percentage of the epithelial intercellular space, a threshold value was applied to separate the empty spaces between the cells from the cellular material. These spaces were then measured and expressed as a percentage of the total epithelial area. Additionally, the thickness of the epithelium was measured using the line tool.

### 2.7. Virus Inoculation

EHV-1 21P40 and EHV-4 VLS 829 were used to test the impact of the SLS-containing supernatant on the equine nasal mucosa. On the other hand, EHV-1 97P70 and EHV-3 04P57 were used to evaluate the outcome of treating the vaginal mucosa with the SLS-containing supernatant. EHV-1 21P40 was isolated from the nasal swab of a Belgian horse in 2021 [30,31]. EHV-4 VLS 829 was obtained from the Office International des Epizooties (OIE) Reference Laboratory (University of Kentucky, Lexington, KY, USA) and was identified by restriction fragment length polymorphism (RFLP) [32]. EHV-1 97P70 was isolated from the lungs of an aborted equine fetus in 1997 [31,33]. EHV-3 04P57 was obtained from a horse with typical ECE lesions in Belgium in 2004 [26].

Following 1 h treatment with CM, EGTA, and the SLS-containing supernatant at 10^−2^, 10^−3^, or 10^−4^, one explant per treatment condition was inoculated with 1 mL of inoculum containing 10^6.5^ TCID_50_ of EHV-1 21P40 or EHV-4 VLS 829 for the nasal septum explants and EHV-1 97P70 or EHV-3 04P57 for the vaginal explants. The explants were then maintained at 37 °C and 5% CO_2_ for 1 h. After two rounds of washing with SFM, the explants were repositioned onto their respective gauzes in 6-well plates containing SFM and incubated for 24 h at 37 °C and 5% CO_2_. At 24 h post-inoculation (hpi), the explants were embedded in Methocel^®^, snap-frozen, and stored at −70 °C until further processing. Mock-inoculated explants with only CM were included concurrently [31].

### 2.8. Evaluation of Viral Replication

Cryopreserved explants were processed using a Leica CM1950 Cryostat (Leica Microsystems GmbH, Wetzlar, Germany). Fifty cryosections were cut from each explant at a thickness of 16 µm and subsequently fixed in 100% methanol at −20 °C for 20 min. First, endogenous biotin was blocked using the Avidin/Biotin Blocking Kit (Invitrogen, Waltham, MA, USA). Subsequently, biotinylated equine polyclonal anti-EHV-1 IgG antibodies (0.15 mg/mL) [34] were used to reveal the EHV-1 and EHV-4 antigens, while biotinylated rabbit polyclonal anti-EHV-3 IgG antibodies (0.15 mg/mL) were used to reveal EHV-3 antigens. Then, streptavidin–FITC^®^ (1/200; Molecular Probes, Eugene, OR, USA) was added. Afterwards, the cryosections were incubated for 1 h at 37 °C. Nuclei were counterstained with Hoechst 33342 (1:100; ThermoFisher Scientific) for 10 min. Between the two staining steps and at the end, the cryosections were washed three times with PBS. Finally, the cryosections were mounted with glycerin DABCO (Janssen Pharmaceutica, Beerse, Belgium) [34]. The stained cryosections were analyzed using a Leica TCS SP2 laser scanning spectral confocal system (Leica, Wetzlar, Germany), using an argon 488 nm laser line to excite FITC and a 461 nm laser line to excite Hoechst 33342. The number of plaques per 4 mm^2^ explant and the average plaque diameter induced by each virus under each treatment condition were determined.

### 2.9. Statistical Analysis

Significant differences (*p* ≤ 0.05) between the SLS-containing supernatant treatments or EGTA treatment and the CM treatment were identified by one-way analysis of variance (ANOVA) followed by Dunnett’s post hoc test. The assumptions of ANOVA were assessed by Levene’s test for the homoscedasticity of the variables and the Shapiro–Wilk test for the normality of the residuals. If homoscedasticity was not met, log transformation of the data was performed prior to ANOVA. When the variable’s normality or homoscedasticity was not met after log transformation, a Kruskal–Wallis test was performed, followed by Dunn’s post hoc test. Moreover, descriptive statistics, including the mean and standard deviation, were computed. IBM SPSS Statistics for Mac, version 29.0 (IBM Corp, Armonck, NY, USA), was used for the analysis. For each parameter, the data of three replicates were plotted as the mean with standard deviation (SD) in dot plot graphs using Prism 9 for MacOS, version 9.3.0. Asterisks were used to denote the levels of statistical significance based on the *p*-value (*p* ≤ 0.05 = *, *p* ≤ 0.01 = **, *p* ≤ 0.001 = ***).

## 3. Results

### 3.1. BSA Is Necessary for SLS’ Hemolytic Activity

The hemolytic activity of the filtered supernatant, presumably containing SLS, was identified through the hemolysis induced on inoculated blood agars (Supplementary Figure S1). A zone of partial β-hemolysis was observed in the BSA-supplemented supernatant. On the other hand, no hemolysis was detected in the supernatant prepared without BSA. Moreover, no bacterial growth was present on either of the inoculated blood agars. Together, these results indicate that the filtered supernatant does not contain viable SEZ bacteria but does contain a substance with hemolytic activity on blood agar, active only in the presence of BSA.

### 3.2. Sls-Containing Supernatant Alters Mucosal Viability in a Concentration-Dependent Manner

**Figure 1:**
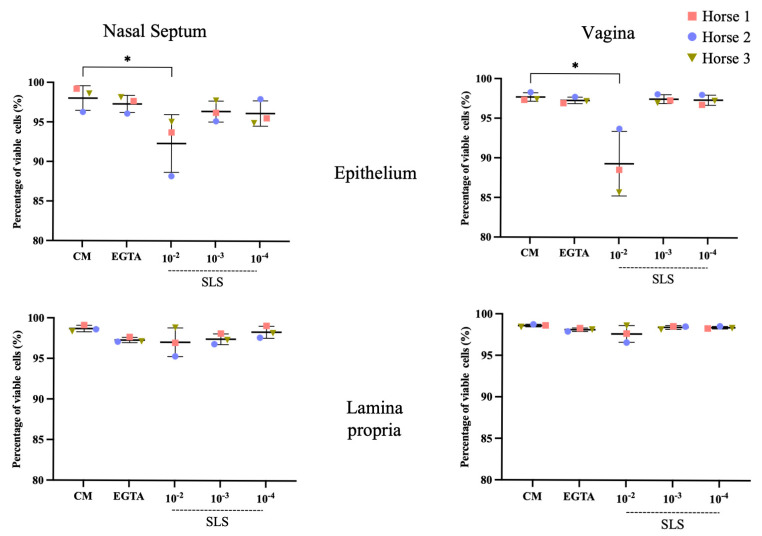
Streptolysin S (SLS) toxin alters epithelial cell viability in a concentration-dependent manner. Cell viability of nasal and vaginal mucosa explants treated for 1 h with co-cultivation medium (CM), ethylene glycol tetraacetic acid (EGTA), and SLS at 10^−2^, 10^−3^, and 10^−4^ concentrations. Terminal deoxynucleotidyl transferase dUTP nick end labeling assay (TUNEL) was performed to detect DNA fragmentation associated with apoptotic cell death. For the nasal explants, one-way ANOVA was performed, followed by Tukey’s post hoc test (*n* = 3 biological replicates). For the vaginal explants, a Kruskal–Wallis test was performed, followed by Dunn’s post hoc test. Asterisks indicate the levels of statistical significance based on the *p*-value (*p* ≤ 0.05 = *).

The SLS-containing supernatant showed a similar pattern of impact on the epithelia of both nasal and vaginal mucosae (Figure 1). In the nasal septum, there was a significant effect of the SLS-containing supernatant on epithelial cell viability (F = 3.45, df = 4, *p* = 0.05). This was concentration-dependent, with the lowest viability observed at 10^−2^ (92.33 ± 3.64%). At this concentration, the viability was statistically significantly lower compared to the viability in the CM-treated explants (98.03 ± 1.56%, *p* = 0.01). In the lamina propria, the number of TUNEL-positive cells did not show a significant difference (F = 1.73, df = 4, *p* = 0.22) under any treatment condition compared to CM-treated explants (98.69 ± 0.40%), indicating that the cytotoxic effect did not reach the lamina propria at any concentration.

In the vagina, the effect of the SLS-containing supernatant on epithelial cell viability was also significant (F = 11.21, df = 4, *p* = 0.001) and concentration-dependent. The CM-treated explants showed the maximum cell viability (97.69 ± 0.53%). Notably, the SLS-containing supernatant at a 10^−2^ concentration induced a significant increase in the number of apoptotic cells with the lowest viability percentage (89.32 ± 4.07%, *p* = 0.03), indicating cytotoxic effects at this concentration despite no significant difference (*p* = 0.07). EGTA- and SLS-containing supernatant (10^−3^- and 10^−4^)-treated explants showed a level of viability that was nearly identical to that observed in CM-treated explants. In the lamina propria, no notable reduction in cell viability was detected under all treatment conditions (F = 1.90, df = 4, *p* = 0.18) compared to CM-treated explants (98.60 ± 0.16%).

Collectively, these results indicate that CM and EGTA at an 8 mM concentration serve as appropriate negative and positive controls, respectively, for the disruption of the cell–cell junction, as no toxic impact on viability was observed. For the SLS-containing supernatant, the only concentration that had a toxic impact on viability was 10^−2^, while the other concentrations (10^−3^ and 10^−4^) showed effects similar to those observed with CM and EGTA.

### 3.3. Sls-Containing Supernatant Induces a Concentration-Dependent Impact on Intercellular Integrity

**Figure 2:**
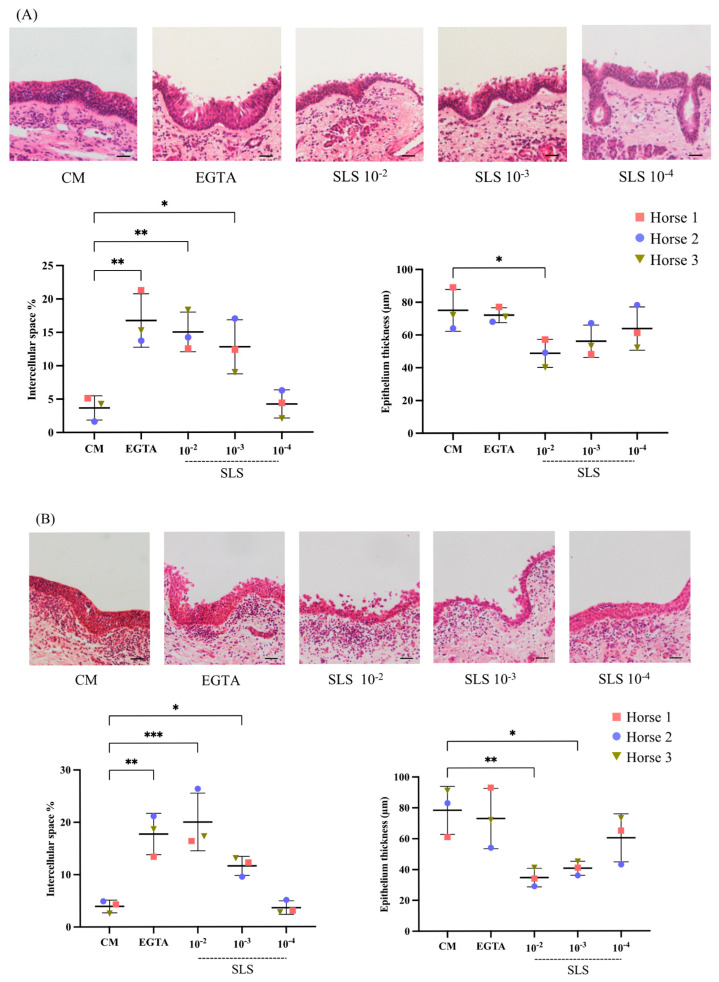
Streptolysin S (SLS) toxin modulates the integrity of the nasal and vaginal epithelium. (**A**) Representative light microscopy images, percentage of intercellular space, and epithelial thickness of nasal explants treated for 1 h with co-cultivation medium (CM), ethylene glycol tetraacetic acid (EGTA), and SLS at 10^−2^, 10^−3^, and 10^−4^ concentrations. (**B**) Representative light microscopy images, percentage of intercellular space, and epithelial thickness of vaginal explants treated under the same conditions. The percentage of the epithelial intercellular space was calculated by measuring the blank spaces after manually determining the region of interest (ROI, the epithelium). The epithelial thickness was measured simultaneously using the line tool function in the Fiji software. One-way ANOVA was performed, followed by Tukey’s post hoc test (*n* = 3 biological replicates). Asterisks indicate the levels of statistical significance based on the *p*-value (*p* ≤ 0.05 = *, *p* ≤ 0.01 = **, *p* ≤ 0.001 = ***). Scale bar: 100 µm.

Figure 2 shows light microscopy images of HE-stained sections of explants treated for 1 h with CM, EGTA, and the SLS-containing supernatant. Additionally, the percentage of the intercellular space and the epithelial thickness were plotted as dot plot graphs.

In the nasal explants (Figure 2A), there was a significant effect of EGTA and the SLS-containing supernatant on the intercellular space (F = 11.58, df = 4, *p* < 0.001) and the epithelial thickness (F = 3.41, df = 4, *p* = 0.05). CM-treated explants showed a baseline value for the intercellular space (3.67 ± 1.81%). This value significantly increased in EGTA and SLS-containing supernatant (10^−2^ and 10^−3^)-treated explants (16.76 ± 3.99% with *p* = 0.002, 15.04 ± 2.97% with *p* = 0.004, and 12.80 ± 4.05% with *p* = 0.016, respectively). The increase in the intercellular space upon treatment with the SLS-containing supernatant decreased with the increasing dilution of the toxin, with the lowest effect observed at 10^−4^, showing a value comparable to that of CM-treated explants. For the epithelial thickness, the opposite trend was observed. The highest thickness was detected in CM and EGTA-treated explants. On the other hand, treatment with the SLS-containing supernatant decreased the thickness, with the lowest value observed at 10^−2^ and a significant difference compared to CM-treated explants (*p* = 0.03).

In the vaginal explants (Figure 2B), the effect was similar to that observed in the nasal explants for both the intercellular space (F = 16.44, df = 4, *p* < 0.001) and epithelial thickness (F = 6.06, df = 4, *p* = 0.01). In CM-treated explants, the intercellular space was 3.93 ± 1.21%, while EGTA treatment significantly increased the intercellular space to 17.74 ± 3.96% (*p* = 0.001). Exposure to the SLS-containing supernatant at 10^−2^ and 10^−3^ concentrations significantly increased the intercellular space to 20.02 ± 5.51% and 11.63 ± 1.85%, respectively (*p* < 0.001 and *p* = 0.049, respectively). The lowest concentration of the SLS-containing supernatant (10^−4^) showed values close to those of CM-treated explants. The thickness measurements showed that CM-treated explants had an average epithelial thickness of 78.33 ± 15.54 μm. EGTA treatment did not significantly alter the epithelial thickness (73.00 ± 19.52 μm) compared to the CM-treated explants. However, treatment with the SLS-containing supernatant at 10^−2^ and 10^−3^ concentrations significantly reduced the epithelial thickness to 34.67 ± 6.03 μm and 40.67 ± 4.51 μm (*p* = 0.009 and *p* = 0.022, respectively). The lowest concentration of the SLS-containing supernatant (10^−4^) caused a moderate reduction in the epithelial thickness (60.33 ± 15.54 µm).

Table 1 presents the percentages of the intercellular space and the corresponding percent changes after 1 h of treatment of nasal and vaginal explants with EGTA and SLS toxin, compared to the control (CM) group. In both nasal and vaginal explants, the notable detachment of epithelial cells was observed following treatment with the SLS-containing supernatant at 10^−2^ and 10^−3^ concentrations, with the exposure of the basement membrane after 10^−2^ treatment at various epithelial locations.

### 3.4. Impact of the Sls-Containing Sez Supernatant on Subsequent Ehv-1 and Ehv-4 Replication in Nasal Explants

**Figure 3:**
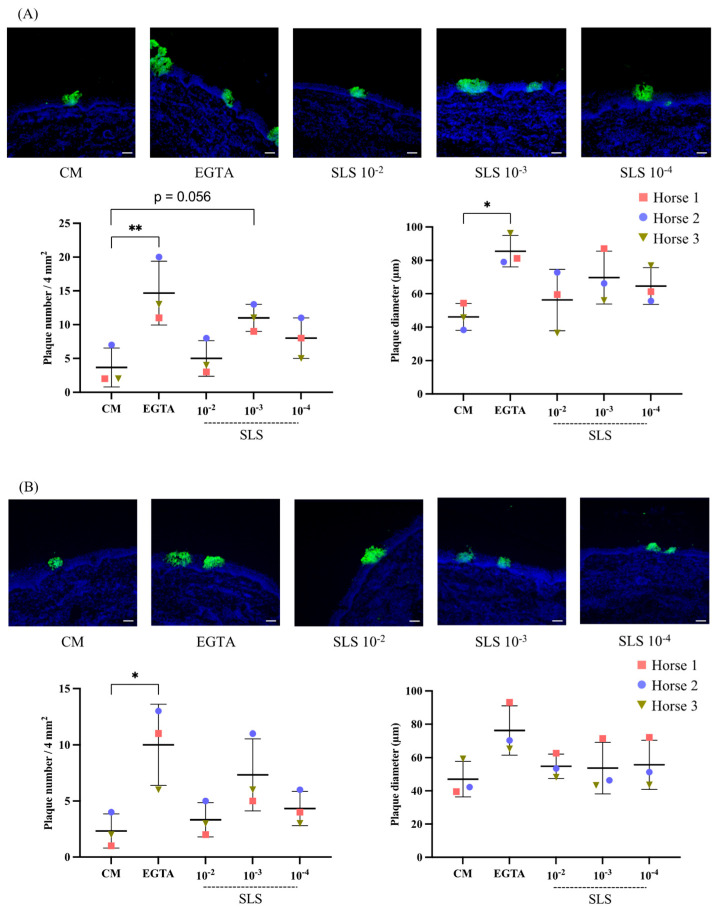
Streptolysin S (SLS) toxin facilitates the replication of equine herpesvirus type 1 (EHV-1) and equine herpesvirus type 4 (EHV-4) in the nasal epithelium. (**A**) Representative confocal microscopy images, number of plaques, and average plaque diameter in nasal mucosa 24 h post-inoculation (hpi) with EHV-1 (10^6.5^ TCID_50_), following 1-h treatment with co-cultivation medium (CM), ethylene glycol tetraacetic acid (EGTA), and SLS at 10^−2^, 10^−3^, and 10^−4^ concentrations. (**B**) Representative confocal microscopy images, number of plaques, and average plaque diameter in nasal mucosa at 24 hpi with EHV-4 (10^6.5^ TCID_50_) after 1-h treatment under the same conditions. EHV-1 and EHV-4 late proteins are shown in green, and nuclei are stained in blue. One-way ANOVA was performed, followed by Tukey’s post hoc test (*n* = 3 biological replicates). Asterisks indicate the levels of statistical significance based on the *p*-value (*p* ≤ 0.05 = *, *p* ≤ 0.01 = **). Scale bar: 50 µm.

Figure 3 shows confocal microscopy images of EHV-1 and EHV-4-induced plaques at 24 hpi in cryosections of nasal explants that were treated for 1 h with CM, EGTA, or the SLS-containing supernatant before inoculation. Moreover, the number and average diameter of the induced plaques were determined per 4 mm^2^ of the explant and are presented in dot plot graphs.

For EHV-1 (Figure 3A), the pretreatment of nasal explants with the preparations (all pretreated groups (EGTA and SLS-containing supernatant combined)) tested in this study significantly impacted both the number of induced plaques (ANOVA; F = 5.93, df = 4, *p* = 0.01) and their diameter (ANOVA; F = 3.79, df = 4, *p* = 0.04) compared to CM-treated explants. The number of plaques in CM-treated explants was 4 ± 3. A statistically significant increase was observed in EGTA-treated explants, with the plaque numbers rising to 15 ± 5 (*p* = 0.006). Treatment with the SLS-containing supernatant at its highest concentration (10^−2^) resulted in plaque numbers similar to those in the CM-treated explants (5 ± 3). Conversely, at the 10^−3^ and 10^−4^ concentrations, the number of plaques increased to 11 ± 2 and 8 ± 3, respectively, with a nearly statistically significant difference at the 10^−3^ concentration (*p* = 0.056). Table 2 presents the percent change in the plaque number for EHV-1 under each treatment condition, relative to CM. The largest average plaque diameter was observed in the EGTA-treated explants (85.48 ± 9.37 µm), with a statistically significant difference (*p* = 0.014). Meanwhile, the average diameter of plaques in CM-treated explants was the lowest (46.18 ± 8.03 µm).

The plaques formed by EHV-4 (Figure 3B) showed a similar pattern in both number (F = 4.91, df = 4, *p* = 0.02) and diameter (F = 2.16, df = 4, *p* = 0.15) to those formed by EHV-1. The lowest number of plaques was recorded in the CM- and SLS-containing supernatant 10^−2^-treated explants (2 ± 2 and 3 ± 2, respectively). In contrast, the EGTA- and SLS-containing supernatant 10^−3^-treated explants had the highest values (10 ± 4 and 7 ± 3, respectively), with a statistically significant difference for EGTA-treated explants compared to CM-treated explants (*p* = 0.011). Table 2 summarizes the percent changes in the plaque number for EHV-4 after each treatment condition, compared to CM. All treatment conditions, except for EGTA, resulted in plaques of a relatively similar diameter. The lowest and highest values were observed in CM- (47.06 ± 10.68 µm) and EGTA-treated explants (76.27 ± 14.80 µm), respectively. However, no significant difference was present.

### 3.5. Replication of Ehv-1 and Ehv-3 in Vaginal Explants Treated with Sls-Containing Sez Supernatant

**Figure 4:**
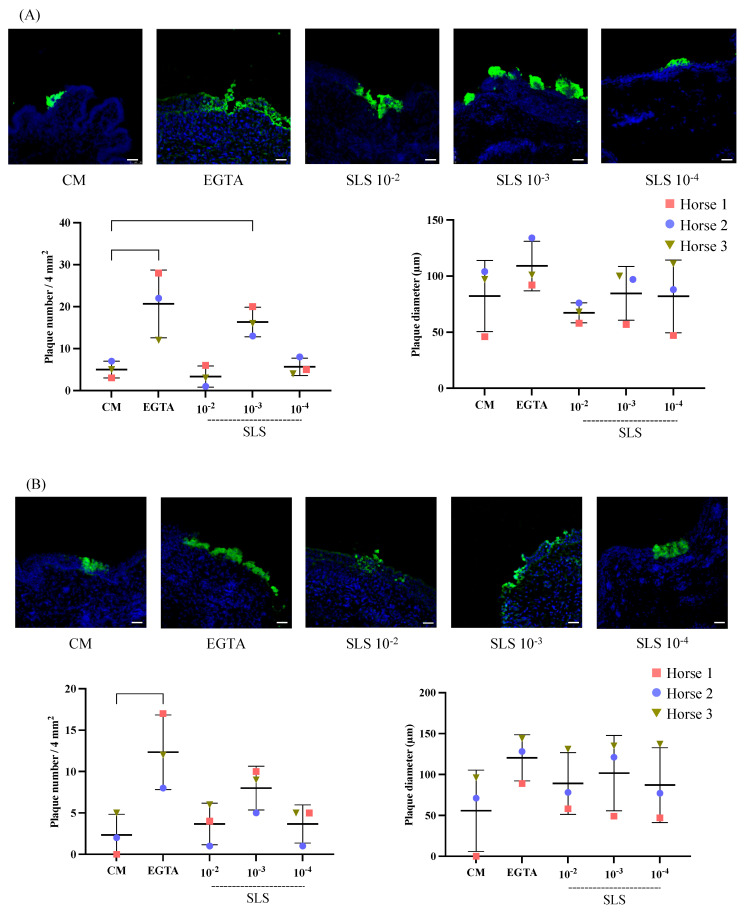
Streptolysin S (SLS) at a 10^−3^ concentration improves the replication of equine herpesvirus type 1 (EHV-1) and equine herpesvirus type 3 (EHV-3) in the vaginal epithelium. (**A**) Representative confocal microscopy images, number of plaques, and average plaque diameter in vaginal mucosa 24 h post-inoculation (hpi) with EHV-1 (10^6.5^ TCID_50_), following 1-h treatment with co-cultivation medium (CM), ethylene glycol tetraacetic acid (EGTA), and streptolysin S (SLS) at 10^−2^, 10^−3^, and 10^−4^ concentrations. (**B**) Representative confocal microscopy images, number of plaques, and average plaque diameter in vaginal mucosa at 24 hpi with EHV-3 (10^6.5^ TCID_50_), after 1-h treatment with CM, EGTA, and SLS at 10^−2^, 10^−3^, and 10^−4^ concentrations. EHV-1 and EHV-3 late proteins are shown in green, and nuclei are stained in blue. One-way ANOVA was performed, followed by Tukey’s post hoc test (*n* = 3 biological replicates). Scale bar: 50 µm.

Figure 4 displays confocal microscopy images of plaques induced by EHV-1 and EHV-3 at 24 hpi, following 1 h of treatment with CM, EGTA, or the SLS-containing supernatant. In addition, the number and average diameter of the induced plaques were determined per 4 mm^2^ explant and are illustrated in dot plot graphs.

For EHV-1 (Figure 4A), the pretreatment of vaginal explants with the substances (all pretreated groups (EGTA and SLS-containing supernatant combined)) included in this study showed an overall significant increase in the induced plaque number compared to CM-treated explants (ANOVA; F = 9.83, df = 4, *p* = 0.002). The number of plaques in CM-treated explants was 5 ± 2. EGTA treatment significantly increased the number to 21 ± 8 (*p* = 0.004). On the other hand, treatment with the SLS-containing supernatant at its highest (10^−2^) and lowest (10^−4^) concentrations resulted in plaque numbers similar to those in the CM-treated explants (4 ± 3 and 6 ± 2, respectively). In contrast, at a 10^−3^ concentration, the number of plaques increased significantly to 17 ± 4 (*p* = 0.029). A summary of the percent change in the EHV-1 plaque number for each treatment, calculated relative to CM, is shown in Table 3. The average plaque diameter showed no significant difference across the treatment groups compared to CM-treated explants (F = 1.06, df = 4, *p* = 0.43). The largest average plaque diameter was observed in EGTA-treated explants (109.00 ± 22.11 µm) but did not significantly differ from that in the CM-treated explants. The smallest plaque diameter was recorded in SLS-containing supernatant 10^−2^-treated explants (67.33 ± 9.02 µm).

The trends in EHV-3 (Figure 4B) were very similar in both the number (F = 5.66, df = 4, *p* = 0.01) and diameter (F = 0.94, df = 4, *p* = 0.48) of induced plaques to those observed with EHV-1. A low number of plaques was identified in CM-treated explants (3 ± 2). Treatment with EGTA significantly increased the number of induced plaques to 13 ± 5 (*p* = 0.007). When exposed to the SLS-containing supernatant at the 10^−2^ and 10^−4^ concentrations, the number of plaques remained low at 4 ± 3. In contrast, treatment with the SLS-containing supernatant at a 10^−3^ concentration resulted in a higher plaque count (8 ± 3), nearly double that of CM-treated explants, although the difference was not statistically significant (*p* = 0.129). Table 3 provides a summary of the percent change in the EHV-3 plaque number under each treatment condition, compared to CM. The average plaque diameter was higher under all treatment conditions compared to CM-treated explants (55.67 ± 49.8 μm); however, no significant difference was observed. The largest average plaque diameters were reported in EGTA- and SLS-containing supernatant 10^−2^-treated explants (120.33 ± 28.3 and 101.67 ± 46.15 μm), respectively.

## 4. Discussion

Due to recent improvements in sequencing technologies and bioinformatics tools, we now have unprecedented capabilities to explore and understand the complexities of microbial communities in much greater detail. In horses, tremendous research conducted over the past decade has shown that distinct microbial populations inhabit the mucosal surfaces of the gastrointestinal [35], respiratory [8], and reproductive tracts [7,36]. The mucosal surfaces represent the portals of entry for various pathogens, including equine herpesviruses. Overcoming the innate barriers that exist at these surfaces is essential in the early stages of pathogenesis, as demonstrated by previous research conducted in our lab [28,37,38,39]. Although virus-to-bacteria priming has traditionally been regarded as the norm, recent studies, as well as our findings, indicate that bacteria-to-virus priming is also a plausible mechanism. Our understanding has significantly advanced regarding the diverse mechanisms by which other pathogens and their products may influence viral processes and behavior [40]. In cattle and pigs, respiratory disease complexes often arise when commensal or opportunistic bacteria colonize the respiratory tract first, with subsequent viral infection triggering severe pathologies [41]. Similarly, in dogs, respiratory viruses are frequently detected in cases of established bacterial pneumonia, suggesting that viral superinfection can exacerbate bacterial disease [42]. These animal studies provided a strong rationale for our experimental design, in which bacterial exposure preceded viral challenge, to better understand the dynamics of co-pathogenesis at the mucosal surface. Despite these efforts, to our knowledge, no studies have examined the impact of toxins produced by commensal bacteria that are part of such microbiomes in horses. For this reason, we investigated the potential effects of SLS from SEZ, the opportunistic pathogen of the equine upper respiratory and lower genital tracts, on EHV infections using two equine explant models. Although explant models cannot fully mimic the dynamic physiological processes, immune responses, and vascular interactions present in vivo, they provide an invaluable tool for preclinical research by closely approximating tissue responses. Therefore, explants provide a better representation of the host compared to cell lines and mouse-based studies.

The SLS toxin is the first identified member of a homologous group of cytolysins known as thiazole/oxazole-modified microcins (TOMMs), which are ribosomally synthesized and post-translationally modified peptides (RiPPs). Advances in bioinformatics have enabled the discovery of SLS and related toxins in other *Streptococcus* species, including human-invasive and β-hemolytic group C and group G streptococci such as *S. dysgalactiae* and *S. anginosus*. In animals, SLS has been identified, so far, in *S. iniae* (fish pathogen) and *S. equi* (horse pathogen). Beyond SLS, TOMMs also include other well-known SLS-like toxins, such as listeriolysin S (LLS) from *Listeria monocytogenes*, clostridiolysin S (CLS) from *Clostridium botulinum* and *C. sporogenes*, and stapholysin S from *Staphylococcus aureus* [43]. The mature SLS peptide is active in the presence of the bacteria (cell-associated SLS). In the absence of bacteria (cell-free SLS), it requires a carrier molecule. Therefore, we used BSA in our study as a carrier and stabilizer for the cell-free SLS. While the precise molecular mechanisms remain elusive, BSA stabilizes the cell-free SLS likely through binding interactions with hydrophobic regions of the toxin and by protecting its labile post-translational modifications from degradation in the extracellular environment, thereby preserving its structural conformation and hemolytic activity in cell-free conditions [24]. Notably, the biochemical characterization of the mature SLS peptide is challenging due to the complex and extensive post-translational modification required for its production, processing, and export. These complexities, in addition to the required coordinated action of multiple enzymes, has historically hindered the recombinant production and purification of active SLS, with no established protocols at the moment [15]. Moreover, the intrinsic instability and lack of immunogenicity further complicate this process [44]. Therefore, it was a limitation in our study that the SLS toxin could not be purified, and molecular techniques, including Western blotting, to confirm the presence of its active and mature forms in the prepared supernatant could not be performed because no commercial antibodies were available [17]. As a result, the assessment of hemolytic activity is widely used to validate the presence of active SLS. In our study, we confirmed the presence of active cell-free SLS by inoculating blood agar with the prepared and filtered supernatant and observing hemolysis without bacterial growth.

Previous studies have acknowledged the role of SLS in tissue damage, which facilitates bacterial translocation and invasion into deeper tissue types. Flaherty et al. demonstrated that the SLS toxin produced by *S. pyogenes* induces programmed cell death and triggers an inflammatory response, leading to tissue damage and the disruption of epithelial barriers and enabling the bacteria to spread into deeper tissue [17]. Similarly, Sumitomo et al. showed that SLS toxin facilitates the movement of *S. pyogenes* by breaking down tight intercellular junctions [18]. In a similar context, Van Cleemput et al. demonstrated that the destruction of epithelial integrity with EGTA or N-acetylcysteine augments the access of EHV-1 to its receptors, which are located basolaterally and concealed by intercellular junctions [28]. Therefore, we hypothesized that SLS could disrupt epithelial integrity in the nasal and vaginal mucosae, thereby facilitating subsequent infection by EHV-1, EHV-4, and EHV-3.

To test our hypothesis, we employed nasal and vaginal explant models to mimic the in vivo environment and study the interplay between SEZ secretions, including SLS, and viral infection. Structural analysis of both the nasal and vaginal epithelia revealed the remodeling impact of SEZ’s SLS-containing supernatant on epithelial integrity, characterized by increased intercellular spaces and decreased epithelial thicknesses, in a concentration-dependent manner, with cytotoxicity observed only at the highest concentration tested (10^−2^). This modulation can be primarily attributed to the disruption of cell–cell and cell–matrix junctional complexes, as previously demonstrated for SLS from Lancefield group A streptococci in the Caco-2, HaCaT, and MDCK cell lines [17,19,45]. Additional mechanisms include alterations in host cell signaling and ion transporters, ultimately leading to significant cytoskeletal changes. Similar observations and interpretations were reported by Van Crombrugge et al., who identified the disruptive effects of α-hemolysin toxin (HLA) from *Staphylococcus aureus* and the adenylate cyclase toxin (ACT) of *Bordetella bronchiseptica* [46]. Moreover, Van Cleemput et al. and Portaels et al. demonstrated the detrimental effects of pollens and *Aspergillus fumigatus* spore proteases, respectively, on epithelial integrity [37,47]. In our study, we could not establish a direct link between SLS and the observed effects due to the presence of multiple toxins in the SEZ supernatant. However, previous research has shown that SLS plays a critical role in tissue damage and specifically contributes to the disruption of intercellular junctions by promoting the cleavage of proteins such as Zonula occludens-1 (ZO-1) and epithelial cadherin (E-cadherin), thereby compromising epithelial barrier integrity [17,18]. Therefore, our findings provide new insights into the potential clinical relevance of SLS activity and the concentrations at which similar effects might occur in the respiratory and reproductive tracts of the host. Further in vivo and clinical studies are needed to better understand these effects.

To further investigate our hypothesis, we conducted experiments using nasal and vaginal explants, focusing on clinically relevant viral infections for each tissue type. For nasal explants, we used EHV-1 and EHV-4, which are important and prevalent respiratory pathogens. Vaginal explants were inoculated with EHV-1 and EHV-3, given their relevance to reproductive health. Treatment with the SLS-containing supernatant at a 10^−3^ concentration enhanced the replication of the tested viruses compared to CM-treated explants. This observation can be attributed to the ability of SLS to impair and disrupt the localization of proteins responsible for forming tight junctions between epithelial cells. These tight junctions are an integral component of innate immunity, sealing the spaces between adjacent epithelial cells, thereby preventing pathogen invasion [48,49]. By modulating these cell–cell junctions, SLS at this concentration (10^−3^) likely facilitates viral access to cellular receptors, thereby increasing the epithelial susceptibility to infection. This facilitation was observed across all viruses used in this study, despite the limitations—most notably, the unavailability of a pure SLS toxin. Similarly, our laboratory has previously shown that disrupting cell–cell junctions in the respiratory tract enhances EHV-1 infection, using agents such as EGTA, HLA toxin, ACT toxin, pollens, and *A. fumigatus* spores [28,37,46,47]. The current study built on this by testing this hypothesis with other EHVs and in both nasal and vaginal explants. The mechanisms by which SLS toxin from SEZ disrupts epithelial integrity to enhance viral infection in equine tissue reflect broader host–pathogen dynamics observed across multiple animal species, particularly in respiratory disease complexes. Comparable dynamics have been documented in respiratory disease complexes in cattle, dogs, and pigs, where respiratory viruses are frequently detected in cases of established bacterial pneumonia [42]. In dogs, SEZ has been implicated in sporadic diseases since the 1970s, with several outbreaks of fetal hemorrhagic pneumonia in recent years [50]. Larson et al. underlined the complex role of SEZ in canine infectious respiratory disease (CIRD) and demonstrated its multifactorial nature, with an emphasis on the canine influenza virus and SEZ co-infection [51]. Similarly, porcine respiratory disease complex (PRDC) often arises when commensal bacteria colonize the respiratory tract first, with subsequent viral infection triggering severe pathologies [20].

On the other hand, this study revealed that the SLS-containing supernatant at 10^−2^ and 10^−4^ concentrations did not affect the rate of viral replication. At the higher concentration (10^−2^), the low level of viral replication was likely due to the cytotoxic effect, which compromised epithelial cell viability. This extensive cellular damage presumably diminished the susceptibility of the cells to the virus by interfering with the entrance of the virus into the cells, as well as the machinery needed for viral replication. In contrast, at the lower concentration (10^−4^), the minimal effect on viral replication can be attributed to the insufficient toxin concentration, which caused only the mild disruption of cellular integrity, without significantly affecting cell viability. Inter-animal variability is a crucial aspect in assessing the biological impacts. Here, the explant model prevailed by giving the ability to test all pretreatment conditions included in this study within one animal. This minimized the inter-animal variability and the number of lab animals needed to test our hypothesis. Additionally, it shows better relevance to the host of SEZ. Therefore, using tissue from three horses, considering this sophisticated approach, provided enough data to perform a statistical analysis.

These results underscore the importance of understanding the equine microbiome and the consequences of microbiome imbalance on the integrity of the respiratory and genital epithelia, as well as the susceptibility to viral infections. This research opens up new avenues for the exploration of the complex risk factors that contribute to the progression of equine herpesvirus infections. Our study primarily examined both the early effects of SLS toxin and viral replication at 24 hpi; consequently, longer-term impacts remain unknown. Therefore, future experiments are encouraged to include extended timepoints, such as 48 and 72 hpi, to characterize the kinetics of viral replication and tissue responses more comprehensively over a longer timeframe. Additionally, the disruption of intercellular junctions, proposed in this study, could be substantiated in future investigations by the immunofluorescence staining of tight junction proteins such as E-cadherin, ZO-1, and claudin-1. Furthermore, and most notably, future in vitro and in vivo studies using SEZ mutants lacking SLS and recombinant SLS are needed to validate the effects of SLS on host mucosal surfaces and to assess its influence on concurrent viral infections. Moreover, the availability of purified SEZ-derived SLS toxin and reliable methods for its detection and quantification are essential to support and extend the findings of this study.

## 5. Conclusions

Our findings demonstrate that a supernatant containing SLS, a toxin produced by the commensal bacterium SEZ, can significantly alter the structure and function of nasal and vaginal epithelial barriers. This alteration, characterized by increased intercellular spaces and reduced epithelial thicknesses, creates a more permissive environment for the entry and replication of equine herpesviruses. It is important to acknowledge that, in the absence of a purified SLS toxin or an SEZ mutant lacking *sag* genes, it is difficult to definitively attribute the observed effects exclusively to SLS. In conclusion, this study adds to existing evidence from previous research on other toxins, highlighting the complex interplay between bacterial toxins, epithelial integrity, and viral infections in the equine respiratory and reproductive tracts.

## Figures and Tables

**Table 1:** Percent changes in the intercellular space of the nasal and vaginal epithelia after EGTA and SLS toxin treatment compared to CM control group.

Condition	Nasal Septum		Vagina	
	Intercellular Space	Percent Change (%)	Intercellular Space	Percent Change (%)
CM	3.67 ± 1.81	0	3.93 ± 1.21	0
EGTA	16.76 ± 3.99 **	+357	17.74 ± 3.96 **	+352
SLS 10^−2^	15.04 ± 2.97 **	+310	20.02 ± 5.51 ***	+409
SLS 10^−3^	12.80 ± 4.05 *	+249	11.63 ± 1.85 *	+196
SLS 10^−4^	4.23 ± 2.11	+15	3.65 ± 1.29	−7

Data are presented as mean of the intercellular space percentage with the standard deviation from three biological replicates. Percent change was calculated relative to the co-cultivation medium (CM) group. Abbreviations: CM, co-cultivation medium; EGTA, ethylene glycol tetraacetic acid; SLS, streptolysin S. Asterisks indicate the levels of statistical significance based on the *p*-value (*p* ≤ 0.05 = *, *p* ≤ 0.01 = **, *p* ≤ 0.001 = ***).

**Table 2:** Percent changes in EHV-1 and EHV-4 plaque numbers in nasal explants after EGTA and SLS toxin treatment compared to CM control group.

Condition	EHV-1		EHV-4	
	Plaque Number	Percent Change (%)	Plaque Number	Percent Change (%)
CM	4 ± 3	0	2 ± 2	0
EGTA	15 ± 5 **	+275	10 ± 4 *	+400
SLS 10^−2^	5 ± 3	+25	3 ± 2	+50
SLS 10^−3^	11 ± 2	+175	7 ± 3	+250
SLS 10^−4^	8 ± 3	+100	4 ± 2	+100

Data are presented as mean plaque number with standard deviation from three biological replicates. Percent change was calculated relative to the co-cultivation medium (CM) group. Abbreviations: EHV-1, equine herpesvirus type 1; EHV-4, equine herpesvirus type 4; CM, co-cultivation medium; EGTA, ethylene glycol tetraacetic acid; SLS, streptolysin S. Asterisks indicate the levels of statistical significance based on the *p*-value (*p* ≤ 0.05 = *, *p* ≤ 0.01 = **).

**Table 3:** Percent changes in EHV-1 and EHV-3 plaque numbers in vaginal explants after EGTA and SLS toxin treatment compared to CM control group.

Condition	EHV-1		EHV-3	
	Plaque Number	Percent Change (%)	Plaque Number	Percent Change (%)
CM	5 ± 2	0	3 ± 2	0
EGTA	21 ± 8 **	+320	13 ± 5 **	+333
SLS 10^−2^	4 ± 3	−20	4 ± 3	+33
SLS 10^−3^	17 ± 4 *	+240	8 ± 3	+167
SLS 10^−4^	6 ± 2	+20	4 ± 3	+33

Data are presented as mean plaque number with standard deviation from three biological replicates. Percent change was calculated relative to the co-cultivation medium (CM) group. Abbreviations: EHV-1, equine herpesvirus type 1; EHV-3, equine herpesvirus type 3; CM, co-cultivation medium; EGTA, ethylene glycol tetraacetic acid; SLS, streptolysin S. Asterisks indicate significant differences (*p* ≤ 0.05). Asterisks indicate the levels of statistical significance based on the *p*-value (*p* ≤ 0.05 = *, *p* ≤ 0.01 = **).

## Data Availability

All data that support this study are included.

## Supplementary Materials

The following supporting information can be downloaded at: https://www.mdpi.com/article/10.3390/v17070980/s1, Figure S1: Assessment of the cell-free SLS hemolytic activity on blood agar.

## Abbreviations

The following abbreviations are used in this manuscript:

%	Percent
°C	Degrees Celsius
ACT	Adenylate cyclase toxin
ANOVA	Analysis of variance
BHI	Brain heart infusion
BSA	Bovine serum albumin
Ca^2+^	Calcium
Caco-2	Human colon carcinoma epithelial cell line
CIRD	Canine infectious respiratory disease
CLS	Clostridiolysin S
CM	Co-cultivation medium
CO_2_	Carbon dioxide
DMEM	Dulbecco’s modified eagle’s medium
DNA	Deoxyribonucleic acid
ECE	Equine coital exanthema
EGTA	Ethylene glycol tetraacetic acid
EHM	Equine herpes myeloencephalopathy
EHV-1	Equine herpesvirus type 1
EHV-3	Equine herpesvirus type 3
EHV-4	Equine herpesvirus type 4
FITC	Fluorescein isothiocyanate
h	Hours
HaCaT	Immortalized human keratinocyte cell line
HE	Hematoxylin and eosin
HLA	α-Hemolysin toxin
hpi	Hours post-inoculation
IgG	Immunoglobulin G
kDA	Kilodalton
LLS	Listeriolysin S
MALDI	Matrix-assisted laser desorption/ionization
MDCK	Madin-Darby canine kidney cells
mg	Milligram
min	Minute
mL	Milliliter
mm	Millimeter
NIH	National Institutes of Health
nm	Nanometer
OD	Optical density
OIE	Office International des Epizooties
PBS	Phosphate-buffered saline
PRDC	Porcine respiratory disease complex
RFLP	Restriction fragment length polymorphism
RiPPs	Ribosomally synthesized and post-translationally modified peptides
ROI	Region of interest
rpm	Rounds per minute
RPMI	Roswell Park Memorial Institute
RT	Room temperature
*sag*	SLS-associated gene
SD	Standard deviation
SEZ	*Streptococcus equi* subsp. *zooepidemicus*
SFM	Serum-free medium
SLS	Streptolysin S
TOF MS	Time-of-flight mass spectrometry
TOMMs	Thiazole/oxazole-modified microcins
TUNEL	Terminal deoxynucleotidyl transferase dUTP nick end labeling
U	Units
*v*/*v*	Volume/volume
× *g*	× times the force of gravity
β	Beta
µg	Microgram
µm	Micrometer
