# Supplementary figures and images for: Streptococcus equi subsp. zooepidemicus Supernatant Containing Streptolysin S Alters the Equine Nasal and Vaginal Mucosa, Modulating Equine Herpesvirus 1, 3 and 4 Infections
Source: Viruses. 2025 Jul 14;17(7):980. doi: 10.3390/v17070980 (PMC12299918; doi:10.3390/v17070980)

A)

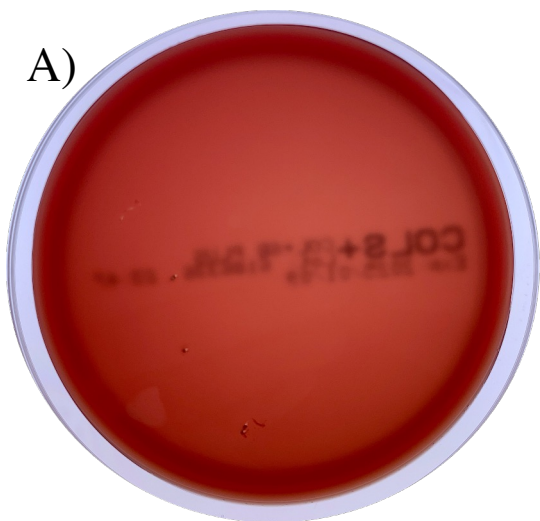

B)

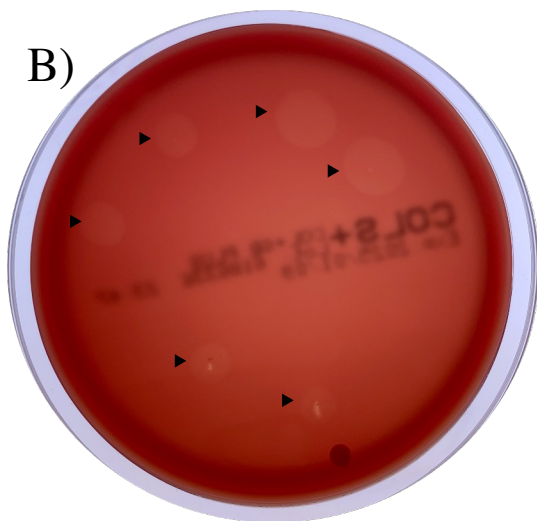

Supplement: Supplementary file 1 [file viruses-17-00980-s001.zip › viruses-3623894-supplementary.pdf]
